# A case of adult ETV6-ASCL6 fusion gene positive chronic myeloproliferative tumor and literature review

**DOI:** 10.12669/pjms.40.11.8556

**Published:** 2024-12

**Authors:** Wei-ge Xu, Pei Wang, Yan-min Yang, Xian-hua Yuan

**Affiliations:** 1 Wei-ge Xu Department of Hematology, First Affiliated Hospital of Xingtai Medical College, Xingtai 054000, Hebei, China; 2 Pei Wang Department of Hematology, First Affiliated Hospital of Xingtai Medical College, Xingtai 054000, Hebei, China; 3 Yan-min Yang Department of Hematology, First Affiliated Hospital of Xingtai Medical College, Xingtai 054000, Hebei, China; 4 Xian-hua Yuan Vice President, First Affiliated Hospital of Xingtai Medical College, Xingtai 054000, Hebei, China

**Keywords:** ETV6-ASCL6, Positive chronic, Myeloproliferative tumor

## Abstract

ETV6-ASCL6 fusion gene plays an important role in hematopoiesis and hematological malignancies, but it is rare in hematological diseases. This paper describes a case of etv6-ascl6 fusion gene positive patient who was successively diagnosed as myelofibrosis, eosinophilic leukemia, basophilic leukemia and secondary acute myeloid leukemia. The clinical manifestations and disease evolution have its unique characteristics.

## INTRODUCTION

A 60 years old male patient with fatigue and palpitation for more than six months, aggravated with dizziness, melena and cough for one week, visited at First Affiliated Hospital of Xingtai Medical College on November 28, 2017. Personal history: drinking for 30 years, Drink about 50-100 ml liquor every day; He had been addicted to smoking for 30 years and smoked about 20 cigarettes a day.

## CASE PRESENTATION

### Physical examination:

Severe anemia, liver not touched, spleen 3-cm below the ribs can be touched. Blood routine test (November 28, 2017): wbc13.89×10^9^ /L, L%69.8%, N%13.5%, M%11%, E%3.3%, B%2.4%, RBC2.31×10^12^/L, HGB60 g/L, PLT70×10^9^/LRet3.8%; Ferritin determination: 529 ng/ml, LDH295 U/L. ultrasound: spleen enlargement; Multiple sites of bone marrow aspiration were dry tap.

Nucleated erythrocytes, young granulocytes and tear drop like erythrocytes can be seen on blood smear. Eosinophils and basophils are easy to see. Bone marrow biopsy pathology (light anterior superior spine): There was obvious increase of eosinophilic cells in bone marrow; Fibrous tissue hyperplasia, reticular fiber staining (mf1-2 grade). Bone marrow biopsy pathology (right anterior superior spine): hematopoietic tissue 90%, adipose tissue 10%, myelodysplastic extremely active. The proportion of granulocyte and erythroid in hematopoietic tissue increased significantly. Granranid precursor cells were occasional, and cells were scattered or visible in pairs at the stages of middle or late, mainly eosinophils, and neutrophils were rare. Erythroid primitive early stage cells were occasionally seen, mainly middle and late stage cells, scattered and occasionally small piles.

Megakaryocytes increased slightly, 6-10 cells / GPF, and were multilobular nuclei. Focal hyperplasia of fibrous tissue. Bone marrow biopsy diagnosis was idiopathic hypereosinophilic syndrome with focal fibrosis of bone marrow. FIP1L1/PDGFRα ETV6-DGFRβBCR / ABL (210), BCR / ABL (190) and FGFR1 were negative. MPN related gene mutation screening test report: ASXL1 gene coding sequence found; The mutation frequency of p. R916X was 49.0%; The mutation frequency of p. Q123X was 47.9%; mutation of p.S507G was found in EP300 gene coding sequence with mutation frequency of 51.6%; A mutation of p.T84M was found in KIT gene coding sequence, and the mutation frequency was 50.8%. CALR, JAK1, JAK2 and MPL were negative. Primary diagnosis was primary myelofibrosis. On December 27, 2017, he visited “Institute of Hematology, Chinese Academy of Medical Sciences” for further diagnosis and treatment.

The hospital considered a diagnosis of “eosinophilia syndrome” and gave symptomatic treatment. But there was no improvement in the blood cells. 20 days later, reexamination of bone marrow (sternum) showed that “Bone marrow triplet hyperplasia with primitive cells is more visible (Raw granulocytes were generated at 9.0%), the proportion of basophils is increased (20%), the mature erythrocytes are heteromorphic; Bone marrow biopsy pathology (iliac bone) Bone marrow hyperplasia was extremely active (> 90%), the proportion of granulocyte red increased significantly. Granulocyte cells are mainly alkalophilic and acidophils, with more cells in the early stage; Reticular fiber staining (MF grade 2-3).

### Immunohistochemistry:

CD34-, CD117-, MPO minority +, Lysozyme minority +, CD5 minority +, CD3 minority+, CD15 minority +, CD42b megakaryocytes +, PAX5-, CD20-, CD123 minority +. Blood samples were collected in all cases under fasting condition in the morning.T-lymphocyte subsets in peripheral blood were determined by flow cytometry.

### Chromosome

Poor cell proliferation. Flow cytometry: The proportion of myeloid primitive cells is too high, with abnormal phenotype. Peripheral blood smear: basophils 32%. Blood routine test: wbc19.21×10^9^/L, HGB56 g/L, PLT38×10^9^/L. Decitabine monotherapy (10 mg, d 1,3,5,7,9) was administered in First Affiliated Hospital of Xingtai Medical College in April 2018. The leukocytes decreased slightly after chemotherapy, but soon the peripheral blood leukocytes and basophils increased gradually again. In May 2018, blood routine showed WBC 27.52×109/L, HGB43 g/L, PLT2×109/L; Bone marrow images showed decreased nucleated cell proliferation, with 1.2% primitive cells and 47.20% basophils; Peripheral blood smear: basophils 75.0%; Bone marrow biopsy: extensive proliferation of fibrous tissue in bone marrow, eosinophils were easily seen, reticular fiber staining (MF-3 grade).The chemotherapy regimen was adjusted to low- dose HA combined with decitabine (HHT 1 mg d1-10, Ara-C 25 mg q12 h d1-14, DAC 10 mg D1, 3, 5, 7, 9) chemotherapy. Blood routine after chemotherapy (2018-8-3): WBC4.54×109/L, HGB69.00 g/L, PLT16×109/L. Peripheral blood smear: basophils 33.0%. Bone marrow smear: basophils 30%. Blood routine test (September 4, 2018): wbc15.63×109/L, HGB52.20g/L, PLT16×109/L; Renal function: crea 413 umol/L, ua 571 umol/L; Coagulation function: aptt42.60 s, pt21.10 s, fib1.10 g/l, tt22.80 s. The patient’s general condition gradually deteriorated, poor diet, severe bleeding symptoms (oral and urinary bleeding), severe anemia, thrombocytopenia, severe pulmonary infection, renal insufficiency, splenomegaly, progressive aggravation, splenic infarction, abdominal distension, and massive ascites.

Support treatment was given and chemotherapy was postponed. One month later, the white blood cells increased progressively. With an intermittent infusion of red blood cells and platelets, blood routine examination (September 18, 2018): white blood cell: 124.3x109/L, hemoglobin: 47.7 g/l, platelet: 40x109/L. Considering that the patient could not tolerate regular chemotherapy, he was given palliative treatment of leucocyte reduction with hydroxyurea. The patient developed intermittent headache, hearing loss in the right ear and double shadow of vision on September 21, 2018. Blood routine examination: white blood cell: 101.22x109/L, lymphocyte count: 28.85x109/L, neutrophil count: 60.74x109/L, eosinophil count: 2.32x109/L, basophil count: 3.84x109/L, hemoglobin: 43.00 g/l, platelet: 24x109/L. CT of head: left cerebellar nodular high-density lesions, new than before. Central infiltration of tumor cells was considered. Continue to give hydroxyurea leucocyte and blood transfusion products, hemostasis, nutritional support treatment. At the same time, the proportion of primitive cells and eosinophils increased gradually. Blood routine test (September 29, 2018):

WBC 78.31x109/L, Hgb 56.30 g/l, PLT 16x109/L; Peripheral blood smear: the whole piece of white blood cells was too much, neutral lobulated nucleus 25%, acidophilic 37%, basophilic 31%, lymphocyte 7%. Peripheral blood NGS fusion gene detection results (Youxun): MLL-PTD positive, ETV6-ASCL6 positive (fusion abundance was 4.52%). However, the general condition of the patient was very unsatisfactory, and no chemotherapy drugs was given, only the treatment was supported. Blood routine test (November 5, 2018): WBC 52.81x109/L, Hgb 70.60 g/l, PLT 33x109/L; Peripheral blood smear: the size of mature red blood cells did not change significantly. The classification of white blood cells was as follows: primitive cells 23%, lobulated nuclei 9%, acidophilic 34%, basophilic 25%, lymphocytes 8%, monocytes 1%; The patient gradually developed lethargy and progressive aggravation of multiple organ failure. On December 10, 2018, he died.

## DISCUSSION

In this case, the patient appeared clinically like a myeloproliferative tumor with an ETV6-ASCL6 fusion gene. ETV6-ASCL6, resulting from t (5; 12) (q31; p13), and associated with myeloproliferative disease, MDS or acute myeloid leukemia with eosinophilia or basophilia, can act as an independent pathogenic gene and also often present together with other chromosomal abnormalities or complex karyotypes.^1^ At present, about 23 patients with hematological diseases with t (5; 12) (q31; p13) have been reported, of which more than 10 patients have been verified as ETV6-ASCL6 fusion by F T S H and RT- PCR, mainly for patients with myeloid tumors including aCML, MDS, AML, and non- specific chronic eosinophil leukemia (CEL-NOS). In more than 10 patients, there were associated with eosinophilic and / or basophil abnormalities, which were the majority of eosinophil abnormalities. In one case report of ETV6-ASCL6-positive AML, we observed the conversion of elevated eosinophils to basophils, suggesting that is the marker of the progression of the disease.^2^ Male patients were more common, with a mean age of 47 years (27-68 years) and a mean survival period shorter than one year.

ETV6 is a member of the ETS family of transcription factors. It has been widely studied in hematological malignancies because of its fusion with other chromosomal segments.^1^,^3^ It contains eight exons. So far, more than 20 translocations related to ETV6 gene have been reported, such as runxl, PDGFRb, ABL, JAK2, ntrk3, ascl6, etc. However, the pathogenesis of ETV6 has not beendetermined yet.^4^ The most commonly discussed are:

The translocation of ETV6 gene with receptor / non-receptor tyrosine kinase genes, such as platelet-derived growth factor PDGFRb. At present, the reported cases of MDS, MPN and CML with ETV6 gene positive showed varying degrees of eosinophilia, and some patients were effective in TKI treatment. Most scholars speculate that it is related to this mechanism.

The ETV6 gene translocations with transcription factors and other protein molecules. For example, the RUNX l gene is the second gene found to be rearranged with ETV6 after PDGFRB, and the ETV6 / RUNXl fusion gene is considered the most common fusion gene in early childhood acute lymphoblastic leukemia. Research found that the ETV6 / RUNXl fusion gene is in childhood ALL and associated with the prognosis.^5^ The ETV6 / RUNXl-like fusion gene was also incorporated into the genotyping of B-ALL. It is less common in adult ALL. The First Affiliated Hospital of Soochow University reported a patient with IL3-ETV6-positive CEL-NOS, with a very high mRNA expression level of IL3, which may be the cause of stimulating eosinophil proliferation, but the treatment with tyrosine kinase inhibitor and the treatment with glucocorticoids, hydroxyurea and interferon was ineffective.^6^

Promoter activation of the ETV6 gene causes aberrant oncogene expression. For example, the ETV6 / EVIl fusion gene, occasionally visible in the MDS and CML-AP stage, is caused by t (3:12). ETV6 did not retain any domain in this fusion gene with only exons one and two. It has been shown that EVI1 gene overexpression can induce leukemia in mice, so it is speculated that the promoter of ETV6 gene is working, making the EVIl gene overexpression.

In recent years, people’s interest in chromosomal abnormalities t (5; 12) has increased dramatically. The t (5; 12) (q31-33; p12-13), caused eosinophilia and monocytosis or myeloid primitive ocytosis, and responded to hydroxyurea or imatinib, but was also resistant and rapidly deteriorated.^7^ These characteristics are believed to be due to activation of the platelet-derived growth factor receptor (PDGFRb) gene rearrangement located at chromosome band 5q31-33. Gene fragment ectopic in these regions has been occasionally found in acute lymphoblastic leukemia,^8^ mainly Pre-B-ALL, which is currently believed to occur and develop similar to myeloid leukemia.

The common breakpoints of etv6-ascl6 fusion were exons one and two of ETV6 gene. It will not enhance the tyrosine kinase activity of the fusion protein. The current case reports also found that etv6-ascl6 fusion patients are ineffective in the treatment of tyrosine kinase inhibitors.^9^ Hydroxyurea combined with cytarabine can be used for combination therapy, and bone marrow transplantation can be carried out after remission.^10^ In this case, the reaction to demethylation and hydroxyurea treatment was very poor, and the condition deteriorated rapidly. At the onset of the disease, eosinophils in bone marrow and peripheral blood of the patient were mainly elevated, which quickly turned to basophils. In the late stage of the disease, the proportion of primitive cells and eosinophils and basophils increased. At the same time, severe secondary bone marrow fibrosis was found in the whole process.

The process of disease transformation and deterioration and invasion were the first cases, and the specific mechanism was still unclear. The combination chemotherapy of hydroxyurea and decitabine failed to prolong the survival of patients. The survival time from diagnosis to death was less than one year.

### Limitations:

It includes small number of cases. This case sharing and research was a rare case, and attention should be paid to summarizing in clinical practice. In the future, relevant genetic tests should be improved for similar cases in order to identify and summarize patients of the same type.

## CONCLUSIONS

ETV6-ASCL6 fusion gene screening is recommended for patients with atypical chronic myeloid leukemia, secondary AML, and refractory eosinophilia, in order to judge the prognosis and accumulate more treatment experience.

### Authors’ Contributions:

**WX** and **PW:** Carried out the studies, participated in collecting data, and drafted the manuscript, and are responsible and accountable for the accuracy or integrity of the work.

**YY:** Performed the statistical analysis and participated in its design.

**XHY:** Participated in acquisition, analysis, or interpretation of data and draft the manuscript.

All authors read and approved the final manuscript.
